# Impact of housing condition on indoor-biting and indoor-resting *Anopheles arabiensis* density in a highland area, central Ethiopia

**DOI:** 10.1186/1475-2875-12-393

**Published:** 2013-11-05

**Authors:** Abebe Animut, Meshesha Balkew, Bernt Lindtjørn

**Affiliations:** 1 Center for International Health, University of Bergen, Bergen, Norway; 2 Aklilu Lemma Institute of Pathobiology, Addis Ababa University, PO Box 1176, Addis Ababa, Ethiopia

## Abstract

**Background:**

Exposure of individuals to malaria infection may depend on their housing conditions as houses serve as biting and resting places of vectors. This study describes the association of housing conditions with densities of indoor-biting and indoor-resting *Anopheles arabiensis* in Hobe, Dirama and Wurib villages of a highland area in central Ethiopia.

**Methods:**

Data on housing conditions, including presence of house apertures, number of occupants and number and the type of domestic animal tethered inside, were collected. Indoor-biting mosquitoes were sampled using Centers for Disease Control (CDC) light traps and indoor-resting mosquitoes sampled with pyrethrum spray catches (PSCs) monthly for two years (July 2008 to June 2010). Female anophelines were identified to species and processed. Univariate and general linear estimating equation allowing for repeated measures were used to assess the contribution of housing conditions for indoor-biting and indoor-resting *An. arabiensis*.

**Results:**

About 96% (4,597/4,788) of anophelines were caught inside residential houses. Nine anopheline species were identified, among which *An. arabiensis* was most prevalent (2,489; 52%). Vectors entering houses were higher in those situated at low (β = 4.475; 95% CI = 3.475-5.476; p <0.001; β = strength of the association) and medium (β = 2.850; 95% CI = 1.975-3.724; p <0.001) altitudes compared to high altitude, and where houses have no windows (β = -0.570; 95% CI = -1.047-0.094; p = 0.019) compared with those that have. Numbers of indoor-resting vectors were higher in those situated at low (β = 6.100; 95% CI = 4.571-7.629; p <0.001) and medium (β = 4.411; 95% CI = 2.284-6.537; p <0.001) altitudes compared to high altitudes, and where houses had open eaves (β =1.201; 95% CI = 0.704-1.698; p <0.001) compared with those that had closed eaves.

**Conclusion:**

Housing conditions such as presence of open eaves, absence of window, location at low and mid altitudes, were strong predictors of indoor exposure to *An. arabiensis* bite in a highland area of south-central Ethiopia.

## Background

Malaria affects 68% of the Ethiopian population [1]. Although control efforts brought reduction in malaria-related mortality compared to the previous years [1,2], the disease is still among the top causes of morbidity in the country [3,4]. It is seasonal in most areas below 1,500 m altitude and unstable in areas above 1,500 m [1].

Transmission of the disease depends largely on local topography, climate and land use. It may also be influenced by housing conditions [5-7]. Conditions, such as nearby irrigated land, earth roof, tethering livestock inside, window presence, open eaves, absence of separate kitchen and presence of a single sleeping room, were associated with high incidence of child malaria in northern Ethiopia [8]. In Burkina Faso, children living in mud-roofed houses were at a higher risk of *Plasmodium falciparum* infection compared to those in iron sheet-roofed houses [9]. In The Gambia, eaves were the main routes of *Anopheles gambiae* and *Mansonia* spp. entry [10,11]. Houses with a grass roof were associated with increased malaria risk in Mozambique [12].

The association of poorly constructed houses with high malaria infection risk may result from their suitability to indoor abundance of vectors [9,10,13]. Houses are the principal site where malaria vectors bite and rest [10,11,14], hence improved housing may reduce indoor occurrence and the risk of malaria transmission in Ethiopia. However, housing conditions and their impact on indoor abundance of vectors may vary with respect to geography, socio-economy and individual household factors. This study was undertaken to assess the contribution housing conditions make to indoor-biting and indoor-resting *Anopheles arabiensis* in a highland area of central Ethiopia.

## Methods

### Study area and housing conditions

A longitudinal study on the relationship between housing conditions and number of indoor-biting as well as indoor-resting *An. arabiensis* was undertaken in Hobe, Dirama and Wurib villages of south-central Ethiopia once a month for two years (July 2008 to June 2010). The same villages and houses were used for related studies [15,16].

**Figure 1:**
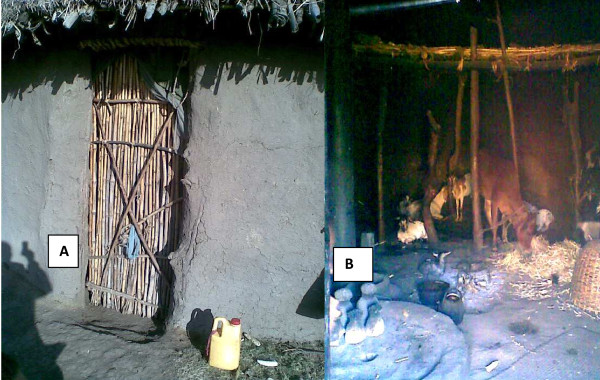
**Typical housing in south-central Ethiopia. A** = door of the house from the outside; **B** = inside the house.

Most of the houses were constructed with mud-plastered wooden walls and grass roofs. They did not have ceilings or separate kitchen. A single living house is used for sleeping, keeping all household belongings, cooking and dining, keeping warm by burning wood and also for tethering domestic animals at night (Figure 1). Data on housing conditions, including presence of house apertures, number of occupants that slept the previous night and number and type of domestic animals tethered indoor the previous night were recorded, while undertaking mosquito sampling, once per month. In addition, the location of each house where mosquitoes were sampled was categorized into either low altitude (Hobe), mid altitude (Dirama) or high altitude (Wurib). The study period was categorized into either dry or wet. Wet were months with average rainfall of greater than 1 mm. They include May, June, July, August, September and October. The number of occupants and domestic animals (cattle, sheep, goat, horse, donkey, and chicken) was recorded by interviewing the head of household or the next elder occupant. House apertures, such as door (unfit or fit), window (absent or present), open eaves (absent or present), hole on wall (absent or present), and hole on roof (absent or present) were recorded by direct observation. All the houses (except one) had unfit doors; therefore the variable door fitness was excluded from the analysis.

### Mosquito sampling

Mosquito sampling was undertaken using Centers for Disease Control (CDC) light trap, pyrethrum spray collection (PSC) and artificial pit shelter (APS) [17] from Hobe, Dirama and Wurib villages. CDC light trap-based collection was made for two consecutive nights inside 20 houses resulting in 40 tap nights per month per village. PSC was made in ten randomly selected houses where no CDC light trap catches was undertaken. Five APSs constructed in shaded areas were used for outdoor-resting mosquito collection in each village. CDC light trap catches were used to collect mosquitoes that attempted to bite humans inside houses during night hours. PSC was used to collect mosquitoes that rest indoors during daylight hours. All female anopheline catches were identified to species, counted and processed, while culicines were discarded after counting. The detailed method is described elsewhere [16].

### Statistics

Indoor- and outdoor-sampled mosquitoes were depicted in a frequency table. Association of each housing condition with the number of either indoor-biting or indoor-resting *An. arabiensis* catches was assessed independently using univariate analysis from which the mean number of *An. arabiensis* catches, including 95% confidence interval (CI) for the mean and significant level was calculated. In the univariate analysis, an independent variable with p value less than 0.1 was considered as a potential predictor and was re-analysed using generalized estimating equation (GEE) multivariate analyses for repeated measures. The dependent variable, number of *An. arabiensis*, fitted to a negative binomial distribution with a log link function [18]. Variables with p values <0.05 in the GEE were considered as strong predictors. Data were analysed using PASW Statistics version 18 (SPSS Inc, Chicago, IL, USA).

### Ethics

The study was ethically cleared by the Ethical Committee of the Faculty of Medicine, Addis Ababa University and The National Health Research Ethics Review Committee (NERC) of Ethiopia with reference number RDHE/48-85/2009. All anopheline collections were undertaken following verbal consent of households.

## Results

A total of 16,894 mosquitoes were sampled of which 71.7% (12, 106/16,894) were culicines and the remaining 28.3% (4,788/16,894) were anophelines (Table 1). Among the total 4,788 female *Anopheles* catches, 96% (4,597) was from inside residential houses. The highest number of anophelines was collected from Hobe (low altitude village) and the lowest from Dirama (mid altitude). *Anopheles arabiensis* was the most common vector in the area (2,489; 52%) followed by *Anopheles demeilloni* (1,261; 26.3%), *Anopheles christyi* (432; 9.02%), *Anopheles pharoensis* (408; 8.52%), *Anopheles cinereus* (166; 3.5%), *Anopheles coustani* (16; 0.33%), *Anopheles culicifacies* (12; 0.25%), *Anopheles garnhami* (3; 0.06%) and *Anopheles rhodesiensis* (1; 0.02).

**Table 1:** Diversity and abundance of anopheline mosquitoes in three villages of central Ethiopia, July 2008-June 2010

**Mosquito**	**Hobe**			**Dirama**			**Wurib**			
	**CDC**	**PSC**	**APS**	**CDC**	**PSC**	**APS**	**CDC**	**PSC**	**APS**	**Total (%)**
*An. arabiensis*	874	1253	19	138	185	0	15	5	0	2,489 (52)
*An. pharoensis*	359	18	0	17	9	0	5	0	0	408 (8.52)
*An. christyi*	7	0	0	23	4	1	312	73	12	432 (9.02)
*An. cinereus*	3	2	0	19	7	0	95	24	16	166 (3.5)
*An. demeilloni*	1	0	0	91	4	2	882	140	141	1,261 (26.3)
*An. coustani*	11	0	0	2	1	0	2	0	0	16 (0.33)
*An. culicifacies*	0	0	0	0	0	0	7	5	0	12 (0.25)
*An. garnhami*	0	0	0	0	0	0	3	0	0	3 (0.06)
*An. rhodesiensis*	0	0	0	0	0	0	1	0	0	1 (0.02)
Total anopheline	1,255	1,273	19	290	210	3	1,322	247	169	4,788 (100)
Total culicine	4,557	578	1,024	988	113	490	1,483	324	2,549	12,106
Total mosquitoes	5,812	1,851	1,043	1,278	323	493	2,805	571	2,718	16,894

CDC = Centers for Disease Control light trap catches; PSC = pyrethrum spray catches;

APS = Artificial pit shelter catches.

Wurib had nine anopheline species while Hobe and Dirama had six species each. *Anopheles arabiensis* was highest in Hobe (2,146) followed by Dirama (323) and Wurib (20). Similar distribution pattern was observed for *An. pharoensis* and *An. coustani*. Catches of *An. christyi*, *An. demeilloni* and *An. cinereus* were highest in Wurib followed by Dirama and very low or scarce in Hobe. From the total 191 outdoor catches, the highest number of anopheline species (n = 169; comprising *An. demeilloni* = 141, *An. cinereus* = 16 and *An. christyi* = 12) was from Wurib while the lowest (n = 3; composed of *An. demeilloni* = 2 and *An. christyi* = 1) being from Dirama. Only one species (*An. arabiensis*; n = 19) was collected from the APS in Hobe.

Table 2 presents housing conditions and associated mean number of *An. arabiensis* catches. Mean number of indoor-biting *An. arabiensis* was significantly higher (p = 0.035) in houses with two or more goats tethered the previous night (mean = 1.06; 95% CI = 0.70-1.42) compared to the houses with less than or equal to one goat (mean = 0.60; 95% CI = 0.38-0.82). Houses with no window had significantly more mosquitoes (mean = 1.02; 95% CI = 0.78-1.27) compared to those with a window (mean = 0.28; 95% CI = -0.04-0.60). Houses with holes on their roof had significantly higher mosquitoes (mean = 1.12; 95% CI =0.75-1.50) compared to the houses with no holes (mean = 0.61; 95% CI = 0.38-0.83). Density of indoor-biting *An. arabiensis* also varied significantly with respect to altitudinal location and was highest in the houses located at the low altitude village (mean = 1.82; 95% CI = 1.53-2.12).

**Table 2:** **Estimation of average number of indoor *Anopheles arabiensis *** **catches per housing conditions using univariate analysis in three villages of central Ethiopia, July 2008-June 2010**

**Housing condition**	**Indoor-biting *An. arabiensis***		**Indoor-resting *An. arabiensis***	
	**Mean (95% CI)**	**P value**	**Mean (95% CI)**	**P value**
**Occupants**				
≤4	0.57 (0.33–0.80)	0.055	1.48 (0.58–2.38)	0.042
≥5	0.91 (0.65–1.18)		3.05 (1.84–4.27)	
**Number of cattle**				
≤2	0.61 (0.34–0.89)	0.429	5.62 (3.56–7.68)	0.004
≥3	0.75 (0.54–0.96)		1.81 (0.29–3.32)	
**Sheep**				
≤1	0.73 (0.49–0.97)	0.921	2.36 (1.44–3.28)	0.402
≥2	0.71 (0.40–1.02)		1.61 (0.12–3.10)	
**Goat**				
≤1	0.60 (0.38–0.82)	0.035	1.97 (1.11–2.83)	0.333
≥2	1.06 (0.70–1.42)		2.99 (1.12–4.87)	
**Horse**				
0	0.70 (0.50–0.90)	0.441	2.21 (1.41–3.01)	0.501
≥1	0.96 (0.33–1.58)		0.73 (-3.51–4.96)	
**Donkey**				
0	0.75 (0.55–0.95)	0.493	2.32 (1.50–3.14)	0.155
≥1	0.56 (0.07–1.06)		0.28 (-2.42–2.97)	
**Chicken**				
≤1	0.62 (0.36–0.88)	0.263	1.91 (0.92 –2.91)	0.459
≥2	0.84 (0.57–1.12)		2.52 (1.26–3.79)	
**Window**				
Absent (n = 157)	1.02 (0.78–1.27)	<0.001	2.35 (1.30–3.39)	0.628
Present (n = 120)	0.27 (-0.04–0.60)		1.95 (0.72–3.18)	
**Hole on roof**				
Absent (n = 210)	0.61 (0.38–0.83)	0.023	1.17 (0.24–2.10)	<0.001
Present (n = 97)	1.12 (0.75–1.50)		4.81 (3.31–6.31)	
**Holes on wall**				
Absent (n = 138)	0.67 (0.32–1.02)	0.628	0.73 (-0.48–1.94)	0.002
Present (n = 171)	0.77 (0.54–1.01)		3.27 (2.22–4.32)	
**Open eaves**				
Absent (n = 198)	0.66 (0.43–0.88)	0.160	0.77 (-0.15 –1.69)	<0.001
Present (n = 98)	0.97 (0.60–1.34)		5.67 (4.22–7.12)	
**Village**				
Low	1.82 (1.53–2.12)	<0.001	5.35 (4.14–6.57)	<0.001
Mid	0.30 (-0.002–0.598)	<0.001	0.83 (-0.42–2.08)	<0.001
High	0.03 (-0.263–0.315)		0.02 (-1.17 –1.21)	
**Season**				
Wet	0.65 (0.34–0.96)	0.271	1.23 (-0.06–2.51)	0.023
Dry	0.89 (0.61–1.17)		3.22 (2.08–4.37)	

Mean number of *An. arabiensis* resting in houses where greater than or equal to five occupants slept the previous night (mean = 3.05; 95% CI = 1.84-4.27) was significantly higher (p = 0.042) than in those with less than or equal to four occupants (mean = 1.48; 95% CI = 0.58-2.38). The mean number of mosquitoes in houses where less than or equal to two cattle tethered the previous night (man = 5.62; 95% CI = 3.56-7.68) was also significantly higher (p = 0.004) than the number in houses where greater than or equal to three cattle tethered (mean = 1.81; 95% CI = 0.29-3.32). Density of mosquitoes in houses with hole on their roof (mean = 4.81; 95% CI = 3.31-6.31), with hole on wall (mean = 3.27; 95% CI = 2.22-4.32) and with open eaves (mean = 5.67; 95% CI = 4.22-7.12) was significantly higher than in those with no hole on roof (mean = 1.11; 95% CI = 0.18-2.05), with no hole on wall (mean = 0.73; 95% CI = -0.48-1.94) and with no open eaves (mean = 0.76; 95% CI = -0.16-1.69), respectively. Density of indoor-resting *An. arabiensis* either at the low altitude village (mean = 5.35; 95% CI = 4.14-6.57) or the mid (mean = 0.83; 95% CI = -0.42-2.08) was significantly higher than at the high altitude village (mean = 0.02; 95% CI = -1.17-1.21). The number of indoor-resting mosquitoes during the dry season (mean = 3.22; 95% CI = 2.08-4.37) was significantly higher (p = 0.023) than the number during the wet season (mean = 1.23; 95% CI = -0.06-2.51) in the area.

Housing conditions that predict indoor-biting and indoor-resting *An. arabiensis* are presented in Table 3. The number of *An. arabiensis* that bite inside houses located at the low altitude village (Hobe) was 4.475 (95% CI = 3.475-5.476; p <0.001) times relative to the number in the high altitude village. Similarly, the number in the mid altitude village was 2.850 (95% CI = 1.975-3.724; p <0.001) times relative to the high altitude. Houses with window had 57% lower number of indoor-biting *An. arabiensis* (β = -0.570; 95% CI = -1.047-0.094; p = 0.019) relative to those with no window. Similarly, house location at the low or mid altitude village relative to the high altitude and presence of open eaves relative to no open eaves were strong predictors of indoor-resting *An. arabiensis*.

**Table 3:** **Housing condition and indoor abundance of *Anopheles arabiensis *** **based on generalized estimating equation model, south-central Ethiopia, July 2008-June 2010**

**Housing condition**	**Indoor-biting *An. arabiensis***		**Indoor-resting *An. arabiensis***	
	**β (95% CI)**	**p**	**β (95% CI)**	**p**
**Number of occupants**				
≥5	0.010 (-0.559–0.580)	0.278	0.135 (-0.364–0.634)	0.596
≤4	0^*^		0	
**Number of cattle**				
≥3	NA	NA	0.007 (-0.444–0.459)	0.975
≤2	NA			
**Number of goats**				
≥2	-0.027 (-0.498–0.444)	0.530	NA	NA
≤1	0		NA	
**Window**				
Present (n = 120)	-0.570 (-1.047–0.094)	0.019	NA	NA
Absent (n = 157)	0		NA	
**Holes on roof**				
Present (n = 97)	0.289 (-0.368–0.947)	0.388	0.258 (-0.156–0.671)	0.222
Absent (n = 210)	0		0	
**Holes on wall**				
Present (n = 171)	NA	NA	0.243 (-0.241–0.727)	0.325
Absent (n = 138)	NA		0	
**Open eaves**				
Present (n = 98)	NA	NA	1.201 (0.704–1.698)	<0.001
Absent (n = 198)	NA		0	
**Village**				
Low	4.475 (3.475–5.476)	<0.001	6.100 (4.571–7.629)	<0.001
Mid	2.850 (1.975–3.724)	<0.001	4.411 (2.284–6.537)	<0.001
High	0		0	
**Season**				
Dry	NA	NA	0.479 (-0.435–1.393)	0.304
Wet	NA		0	

NA = housing condition not applicable.

The mean number of indoor-biting *An. arabiensis* characterized by feeding status, blood meal source and *Plasmodium* sporozoite infection status with respect to housing condition is presented in Table 4. Houses located in the low altitude village were observed to have significantly highest mean number of fresh fed (2.58), half gravid (0.89), gravid (0.72), unfed (0.75) and bovine fed (1.31) *An. arabiensis* caught by CDC light trap. Houses with no window had higher mean number of fresh fed, unfed, bovine fed, human fed and human and cattle mixed blood fed *An. arabiensis* and the differences were significant.

**Table 4:** **Differences in the mean number of indoor biting *Anopheles arabiensis *** **status (feeding, blood meal source and *Plasmodium *** **infection) with respect to selected housing conditions in three villages of central Ethiopia, July 2008-June 2010**

**Anopheline status**	**Window**		**Village**		
	**Absent**	**Present**	**Low**	**Mid**	**High**
**Fresh fed**					
*Mean*	2.49	0.89	2.58	0.84	0.55
*p*	0.005		0.001		
**Half gravid**					
*Mean*	0.87	0.40	0.89	0.29	0.27
*p*	0.053		0.013		
**Gravid**					
*Mean*	0.68	0.38	0.72	0.25	0.18
*p*	0.196		0.051		
**Unfed**					
*Mean*	0.74	0.29	0.75	0.29	0.27
*p*	0.032		0.034		
**Bovine fed**					
*Mean*	1.34	0.44	1.31	0.56	0.14
*p*	0.018		0.039		
**Human fed**					
*Mean*	1.15	0.61	1.07	0.72	0.57
*p*	0.036		0.234		
**Human and bovine fed**					
*Mean*	0.46	0.08	0.46	0.21	0
*p*	0.008		0.065		
***P. vivax *** **positive**					
*Mean*	0.09	0.03	0.09	0.03	0
*p*	0.299		0.512		
***P. falciparum *** **positive**					
*Mean*	0.01	0	0.01	0	0
*p*	0.346		0.847		

The mean numbers of indoor-resting (caught by PSC) fresh fed, half gravid, gravid, bovine fed, human fed, and human and bovine mixed blood fed *An. arabiensis* were significantly higher in houses having open eaves than in those with no open eaves and also in houses located at either the low or mid altitude village than in the high altitude village (Table 5).

**Table 5:** **Differences in the mean number of indoor-resting *Anopheles arabiensis *** **status (feeding, blood meal source and *Plasmodium *** **infection) with respect to three housing conditions in three villages of central Ethiopia, July 2008-June 2010**

**Anopheline status**	**Open eaves**		**Village**		
	**Absent**	**Present**	**Low**	**Mid**	**High**
**Fresh fed**					
*Mean*	3.03	9.00	8.76	1.87	1.00
*p*	0.001		<0.001		
**Half gravid**					
*Mean*	0.73	2.40	2.22	0.62	0
*p*	0.013		0.031		
**Gravid**					
*Mean*	0.83	2.71	2.64	0.32	0.25
*p*	0.017		0.006		
**Unfed**					
*Mean*	0.20	0.59	0.56	0.10	0
*p*	0.324		0.418		
**Bovine fed**					
*Mean*	1.39	4.14	3.85	1.12	0
*p*	0.001		0.001		
**Human fed**					
*Mean*	1.32	2.90	3.08	0.64	0.33
*p*	0.007		<0.001		
**Human and bovine fed**					
*Mean*	0.27	1.32	1.19	0.16	0.33
*p*	0.002		0.004		
***P. vivax *** **positive**					
*Mean*	0.02	0.04	0.05	0.03	0
*p*	0.538		0.864		
***P. falciparum *** **positive**					
*Mean*	0	0.02	0.02	0	0
*p*	0.310		0.743		

## Discussion

Most *Anopheles* mosquito species in Hobe, Dirama and Wurib villages of central Ethiopia occur inside residential houses. Houses having open eaves, no window, and located at either low or mid altitude village were associated with higher risk of malaria. The indoor occurrence of anophelines in these highland villages could be attributed to several factors among which appropriate indoor microclimate is one [19,20]. The tradition of cooking, sleeping and tethering livestock inside residential houses could contribute to the indoor occurrence of mosquitoes by increasing indoor temperature and providing access to blood meal sources. This in turn contributes to the survival and increased malaria transmission potential of the vectors in the area. Indoor-resting mosquitoes of East Africa are estimated to transmit malaria between 0.3 and 22.5 days earlier than those of outdoor-resting mosquitoes [19]. This study reveals that *An. arabiensis* and *An. pharoensis*, which are malaria vectors in the area [16] and the remaining seven anopheline species, exhibit endophilic behaviour indicating the need to construct mosquito proof houses.

Densities of both indoor-biting and indoor-resting *An. arabiensis* were highest in the low altitude village and decreased with increasing altitude. Similarly, densities of both immature and adult stages of the vector were observed to decrease significantly with increasing altitude in the area during the period [15,16] and so was the risk of acquiring *P. falciparum* and *Plasmodium vivax* malaria [16,21,22]. Density of vectors generally decreases with increasing altitude in highland areas [23].

In this study, houses with open eaves were strongly associated with indoor-resting *An. arabiensis* relative to the houses with no such opening. Eaves could enhance *An. arabiensis* entry to houses and its blood meal sources (human and cattle) which stay indoor during night hours [16] and then rest in the house until oviposition. Houses with open eaves and no ceilings were observed with higher number of *An. gambiae* than those with closed eaves and ceilings [10]. Open eaves were associated with increased risk of *An*. *gambiae s.l.* and *Culex pipiens s.l.* entry in The Gambia [11,24]. *Anopheles gambiae s.s.*, *An. arabiensis*, *Mansonia africana* and *Ma. uniformis* were noted to prefer eaves as the main entry points in Tanzania [25]. The high density of *An. arabiensis* inside houses with open eaves could result from the upward-flying behaviour of the mosquito when encountering wall surfaces and entering houses through these holes having been attracted by microclimatic conditions and odours of humans and cattle coming from the houses [10,11,19,20,26].

This study indicates the need to construct houses with closed eaves, roof and ceilings in Hobe, Dirama and Wurib villages of central Ethiopia in order to minimize indoor-resting *An. arabiensis*, which is the most prevalent and major malaria vector in the area [15,16]. House ceilings made of plywood, synthetic-netting, insecticide-treated synthetic-netting, and plastic insect screen, all installed below open eaves and mud-closed eaves, reduced entry of *An. gambiae* into experimental huts in Gambia [10]. Closing eaves resulted in a three-fold reduction in *An. gambiae s.l.* caught indoors [11]. Eaves screening reduced density of indoor *An. gambiae s.l.*, *Ma. africana* and *Ma. uniformis* significantly in southern Tanzania [25]. Screening houses fully and also equipping them with screened ceilings can reduce indoor exposure to *An. arabiensis* bites as noticed in The Gambia [27] and Kenya [28]. In addition, constructing houses with iron-sheet roof instead of thatched roof may reduce malaria infection risk in south-central Ethiopia as reported from Burkina Faso [9].

The number of *An. arabiensis* that attempted to bite indoors at night was 57% lower in houses with windows than in those with no window. The presence of windows might have increased aeration inside houses, which could reduce indoor temperature. Low indoor temperature in these highland villages could deter the indoor-biting mosquitoes at night. In The Gambia [11], windows and doors were found less important for *An. gambiae* s.l. entry into houses but were the main entry routes of culicines.

*Anopheles arabiensis*, which is the principal malaria vector in Hobe, Dirama and Wurib villages in particular [16] and in Ethiopia in general, was prevalent inside houses located in the low altitude village and in the mid altitude village. Houses with open eaves were also observed to have high density of indoor-resting *An. arabiensis*. Better designed houses and house screens, together with existing malaria control programmes, may help to reduce indoor-biting as well as indoor-resting *An. arabiensis* and hence transmission of the disease significantly.

## Conclusion

Nine species of anopheline mosquitoes, including *An. arabiensis*, which is the primary malaria vector in Ethiopia, were more abundant inside residential houses than outdoors (in pit shelters) in Hobe, Dirama and Wurib village of south-central Ethiopia. Housing conditions such as the presence of open eaves, location at either low or mid altitude village, and absence of windows, were found to be strong predictors of indoor-occurring *An. arabiensis*.

## Competing interests

The authors declare that they have no competing interests.

## Authors’ contributions

AA designed the study, collected data in the field, carried out the data analysis and wrote the first draft of the manuscript. MB participated in the conception of the study, in the study design and editing the manuscript. BL conceived the idea for the study and took part in the study design, data analysis, data interpretation and editing the manuscript. All authors have read and approved the final manuscript.
